# Clinical characteristics of the patients with bacteremia due to *Moraxella catarrhalis* in children: a case–control study

**DOI:** 10.1186/s12879-016-1408-3

**Published:** 2016-02-09

**Authors:** Takanori Funaki, Eisuke Inoue, Isao Miyairi

**Affiliations:** 1 Division of Infectious Diseases, Department of Medical Subspecialties, National Center for Child Health and Development, 2-10-1 Okura, Setagayaku, Tokyo, 1578535 Japan; 2 Center for Clinical Research and Development, National Center for Child Health and Development, 2-10-1 okura, Setagayaku, Tokyo, 1578535 Japan

**Keywords:** *Moraxella catarrhalis*, Bacteremia, Children, Trans-nasal devices, Underlying disease

## Abstract

**Background:**

Invasive diseases due to *Moraxella catarrhalis* are rare in children, even in immunocompromised hosts. Therefore, data regarding clinical characteristics and risk factors of such patients are limited. The aim of this study is to compare the clinical characteristics of patients with bacteremia due to *Moraxella catarrhalis* against those with bacteremia due to *Streptococcus pneumoniae* and *Haemophilus influenzae*.

**Methods:**

We performed a retrospective case–control study to compare patients younger than 18 years of age with positive blood cultures for the three pathogens between June 2008 and May 2014 at our institution. Data regarding patients’ demographics and clinical course were collected from their medical records. Three group comparisons, with *M. catarrhalis* as reference, were made by the Fisher’s exact test and Wilcoxon rank sum test for discrete and continuous variables, respectively.

**Results:**

There were eight cases of *M. catarrhalis*, 110 cases of *S. pneumoniae* (105 patients) and 22 cases of *H. influenzae*. The *M. catarrhalis* group consisted of six females (75 %) with a mean age of 16 months. The majority of patients (7/8, 88 %) had underlying diseases; however, only one was immunocompromised. Characteristically, six patients (75 %) had medical devices including trans-nasal devices (5/8, 63 %). Univariate analysis revealed that underlying conditions (*P* = 0.005), trans-nasal devices (*P* < 0.001), and lower body weight (*P* = 0.016) and low white blood cell count (*P* = 0.011) at the onset of illness were associated with the *M. catarrhalis* group compared to the *S. pneumoniae* group. Meanwhile, the higher rates of the patients with underlying conditions and trans-nasal devices were associated with the *M. catarrhalis* group compared to the *H. influenzae* group (*P* = 0.039, *P* < 0.001, respectively).

**Conclusions:**

The presence of underlying conditions, particularly in those with trans-nasal devices, were characteristic features of patients with bacteremia due to *M. catarrhalis.*

**Electronic supplementary material:**

The online version of this article (doi:10.1186/s12879-016-1408-3) contains supplementary material, which is available to authorized users.

## Background

*Streptococcus pneumoniae*, *Haemophilus influenzae* and *Moraxella catarrhalis* are recognized as the three most common bacterial causes of respiratory infection in children*.* In the pre-vaccine era, *S. pneumoniae* and *H. influenzae* commonly caused invasive diseases including bacteremia and meningitis. However, since the introduction of the pneumococcal conjugate vaccine (PCV) in 2000 and *H. influenzae* type b (Hib) vaccine in 1987 in the United States, the number of patients with invasive pneumococcal diseases (IPD) and invasive haemophilus disease (IHD) has decreased remarkably [1–5]. Similarly, in Japan, the incidence of patients with IPD or IHD has decreased as a result of the introduction of PCV in 2010 and Hib vaccine in 2008 [6]. However, IPD caused by non-vaccine serotype *S. pneumoniae* and respiratory diseases by non-typable *H. influenzae* have since emerged as a source of concern. Similarly, the relative importance of *M. catarrhalis* as a cause of respiratory tract infection such as otitis media, sinusitis and lower respiratory tract disease in otherwise healthy children and elderly persons has been recognized [7]. Invasive diseases due to *M. catarrhalis* are rare in children even in immunocompromised hosts [7, 8]. Hence, data regarding the clinical characteristics and risk factors of patients with invasive *M. catarrhalis* infection are limited. Identification of patients at risk for invasive infection caused by *M. catarrhalis*, is important when considering empiric therapy because most of the strains are beta-lactamase (penicillinase) producers, in contrast to pneumococcus. The aim of this study is to investigate the characteristics of the patients with bacteremia caused by *M. catarrhalis* compared to those with bacteremia caused by *S. pneumoniae* or *H. influenzae*.

## Methods

### Setting and patients selection

We retrospectively identified patients with positive blood cultures for *M. catarrhalis*, *S. pneumoniae* or *H. influenzae* at the National Center for Child Health and Development (NCCHD). The NCCHD is a 490-bed tertiary care children’s hospital in Tokyo, Japan, with approximately 12,000 hospitalizations and 35,000 visits to the emergency department each year. The study period was between June 2008 and May 2014. Patients with a disease onset at ≥18 years of age were excluded.

### Data collection

Blood culture results were extracted from the infectious disease surveillance system (Nippon Becton Dickinson Company, Ltd, Tokyo, Japan) at NCCHD. Clinical data of patients who were included were retrospectively collected using electronic medical records, which were reviewed for: 1) patients’ demographics including age, gender, body weight, underlying medical conditions and diseases, immunization records for PCV and Hib vaccine, medical devices, presence or absence of immunodeficiency, immunosuppressants, and previous antibiotic use; 2) contact with sick person or people, symptoms, laboratory results at presentation, and focus of infection; and 3) management including treatment and disposition, as well as outcome.

### Definitions

The focus of infection was defined based on the clinical diagnosis or simultaneous identification of the pathogen from the suspected focus (e.g. thoracic empyema, arthritis, and meningitis) and the blood. Occult bacteremia was defined based on the criteria from a previous report. [9] Immunodeficiency was defined as follows: 1) primary immunodeficiency including asplenia; 2) immunosuppressant use such as calcineurin inhibitors, steroids (a dosage equivalent to ≥2 mg/kg/day of prednisone or ≥20 mg/day for children who weigh more than 10 kg, for more than 14 days) [10]; and molecular-targeted agents (e.g. rituximab, infliximab); and 3) undergoing or <6 months after completion of cancer chemotherapy. Medical devices included any of the following; 1) central venous catheter (peripherally inserted central catheter, short-term (non-tunneled) central venous catheter, long-term (tunneled) central venous catheter), 2) urinary catheter, 3) intubation (nasotracheal or orotracheal) and tracheostomy, 4) nasogastric tube and elemental diet tube, 5) esophageal thermometer, and 6) other foreign bodies. Onset of disease was defined as community or nosocomial based on 1) whether the patient was hospitalized within 1 month from the onset of illness, and 2) the site at which blood culture sampling occurred (in an outpatient clinic or during hospitalization). Hospital-acquired infection was defined as infection with an onset of illness of more than three days after admission to our hospital. Prior antibiotics use was defined as patients who received antibiotics (oral or intravenous agents) within one week prior to the onset of illness. Duration of fever was defined as the number of days the patient was on antibiotics until defervescence was achieved for more than 24 h without antipyretics. Recurrence was defined as bacteremia or invasive infection due to the same organism within one month of completion of therapy.

### Microbiological methods

Identification of each organism and antimicrobial susceptibility test were performed at our institution. Blood cultures were inoculated into BacT/ALERT® 3D bottles (SYSMEX bioMérieux Co., Ltd., Tokyo, Japan). *M. catarrhalis*, *S. pneumoniae* and *H. influenzae* were identified using standard methods (BD Phoenix™ Automated Microbiology System, Becton, Dickinson and Company, NJ, USA). Antimicrobial susceptibility was determined according to the criteria of the Clinical and Laboratory Standards Institute [11].

### Japanese immunization program

The PCV7 and Hib vaccines became available in Japan in December 2008 on a voluntary basis and became federally funded for children since November 2010. Both vaccines became part of the national immunization program in April 2013. PCV7 was converted to PCV13 in November 2013 [12, 13]. Current immunization schedule recommended by the Japan Pediatric Society is available on the website [14].

### Statistical analysis

Patients’ background and disposition data were summarized by mean, standard deviation (SD), interquartile range and proportion as appropriate. The comparisons of *M. catarrhalis* vs. *S. pneumoniae* and *M. catarrhalis* vs. *H. influenzae* were made by the Fisher’s exact test and Wilcoxon rank sum test for discrete and continuous variables, respectively. We considered a *p*-value of less than 0.05 as a meaningful difference. No adjustment for multiplicity was conducted. The R software (version 3.1.2) [15] was used for statistical calculation.

### Ethics

This study was performed under the institutions opt-out passive consent policy and approved by the ethics committee and the Institutional Board of Privacy and Security at the National Center for Child Health and Development. Patients and guardians wishing to withdraw from the study were able to contact the principal investigator through information provided on notifications publically posted by the institution’s ethics committee. Patients were withdrawn based on verbal request from the patient or the guardian (if patients were under 18 years of age at the time of request). Individual participant data is not reported.

## Results

### Patients/Cases

**Fig. 1:**
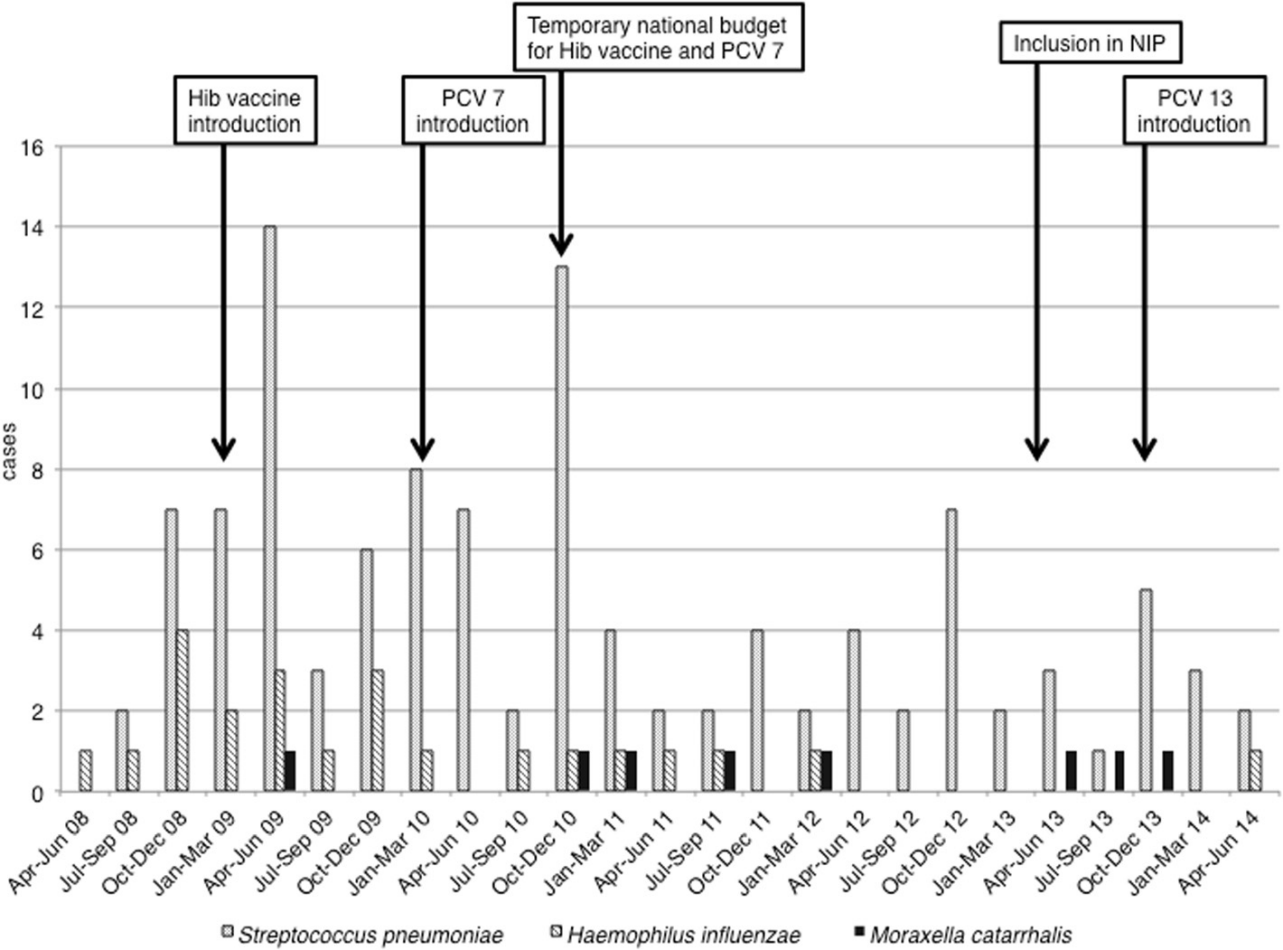
Quarterly number of patients with bacteremia caused by *Moraxella catarrhalis, Streptococcus pneumoniae,* or *Haemophilus influenzae.* X-axis denotes quarterly periods between April 2008 and June 2014. Y-axis denotes the incidence of bacteremia caused by *M. catarrhalis, S. pneumoniae* and *H. influenzae.* Abbreviations: Hib, *Haemophilus influenzae* type b; PCV7, 7-valent pneumococcal conjugate vaccine; PCV13, 13-valent pneumococcal conjugate vaccine; NIP, national immunization program in Japan

Patients included during the study period were as follows: 1) *M. catarrhalis* (*n* = 8), 2) *S. pneumoniae* (*n* = 105 [110 cases]) and 3) *H. influenzae* (*n* = 22). (Figure 1) Pneumococcal bacteremia gradually decreased after federal funding became available for PCV7 and PCV13. Bacteremia due to Hib decreased remarkably after introduction of Hib vaccine with no patients observed after April 2012. One patient developed bacteremia due to non-typable *H. influenzae* in May 2014, while bacteremia due to *M.catarrhalis* occurred sporadically.

### Baseline characteristics

Baseline characteristics among each group are shown in Table 1.

**Table 1:** Patients’ characteristics with bacteremia due to *Moraxella catarrhalis*, *Streptococcus pneumoniae and Haemophilus influenzae*

	*M. catarrhalis*	*S. pneumoniae*	*H. influenzae*	*p*-value*	*p*-value**
	(*n* = 8)	(*n* = 110)	(*n* = 22)		
Age (months, mean ± SD)	16 ± 12	35 ± 32	25 ± 33	-	-
Gender (female, %)	6 (75 %)	51 (46 %)	6 (27 %)	0.153	0.188
Body weight (kg)					
mean ± SD	8.31 ± 2.80	12.49 ± 6.15	10.20 ± 3.10	0.016^a^	0.188
IQR	7.45-9.25	8.70-13.60	8.30-10.88		
Vaccination					
PCV7					
YES (n, %)	6 (75 %)	25 (23 %)	1 (5 %)	0.004^a^	<0.001^a^
PCV13					
YES (n, %)	0 (0 %)	1 (1 %)	0 (0 %)	N/A	N/A
Hib vaccine					
YES (n, %)	5 (63 %)	32 (29 %)	2 (9 %)	0.106	0.007^a^
Underlying diseases					
YES (n, %)	7 (88 %)	39 (35 %)	9 (41 %)	0.005^a^	0.039^a^
Immunodeficiency					
YES (n, %)	1 (13 %)	14 (13 %)	1 (5 %)	1.000	0.469
Immunosuppressants					
YES (n, %)	2 (25 %)	7 (6 %)	0 (0 %)	0.114	0.064
Devices					
YES (n, %)	6 (75 %)	18 (16 %)	0 (0 %)	0.001^a^	<0.001^a^
Trans-nasal devices					
YES (n, %)	5 (63 %)	7 (6 %)	0 (0 %)	<0.001^a^	<0.001^a^
History of previous intubation					
YES (n, %)	5 (63 %)	32 (29 %)	4 (18 %)	0.106	0.032^a^
Contact with sick people					
YES (n, %)	2 (25 %)	45 (41 %)	10 (45 %)	0.472	0.419
Pre-existing antibiotics					
YES (n, %)	0 (0 %)	38 (38 %)	10 (45 %)	0.049^a^	0.029^a^
Hospital acquired infection					
YES (n, %)	6 (75 %)	18 (16 %)	1 (5 %)	0.001^a^	<0.001^a^
Symptoms					
Rhinorrhea (n, %)	2 (25 %)	53 (48 %)	3 (14 %)	0.281	0.589
Cough (n, %)	5 (63 %)	44 (40 %)	5 (23 %)	0.274	0.078
Convulsion (n, %)	1 (13 %)	31 (28 %)	8 (36 %)	0.445	0.374
Vomiting (n, %)	2 (25 %)	14 (13 %)	1 (5 %)	0.296	0.166
Diarrhea (n, %)	1 (13 %)	6 (5 %)	2 (9 %)	0.350	1.000
Body temperature					
Tmax (°C, mean)	38.9	39.3	39.0	0.176	0.557
IQR	38.6–39.2	38.7–40.0	38.5–39.8		
Focus of infection					
Occult bacteremia	0 (0 %)	40 (36 %)	2 (10 %)	0.050	1.000
Pneumonia	4 (50 %)	19 (17 %)	5 (22 %)	0.046	0.195
Meningitis	0 (0 %)	4 (4 %)	8 (36 %)	1.000	0.071
Otitis media/Sinusitis	0 (0 %)	13 (12 %)	1 (5 %)	0.596	1.000
Others	3^b^(38 %)	10^c^(9 %)	5^d^(22 %)	0.048	0.643
Unknown	1 (12 %)	24 (22 %)	1 (5 %)	1.000	0.469
Empiric antibiotic therapy^e^					
Aminopenicillins^f^	0 (0 %)	19 (17 %)	0 (0 %)	0.351	N/A
Beta-lactam/Beta-lactamase inhibitor combinations^g^	3 (38 %)	8 (7 %)	1 (5 %)	0.026	0.048
Second generation cephalosporins^h^	1 (12 %)	2 (2 %)	0 (0 %)	0.191	0.267
Third generation cephalosporins^i^	3 (38 %)	71 (65 %)	17 (77 %)	0.147	0.078
Others^j^	1 (12 %)	17 (15 %)	8 (36 %)	1.000	0.374
No treatment	0 (0 %)	3 (3 %)	2 (9 %)	1.000	1.000
Initiation with combination therapy	0 (0 %)	10 (9 %)	6 (27 %)	1.000	0.155
Duration of fever after treatment (days, mean ± SD)	2.63 ± 0.92	1.86 ± 1.00	3.77 ± 3.61	0.012^a^	0.599
IQR	2–3	1–2	2–4.75		
WBC (/μL, mean ± SD)	14,774 ± 13,750	19,706 ± 7862	17,805 ± 10,173		
IQR	9395–12,158	14,548–24,318	9468–22,988	0.011^a^	0.277
ANC (/μL, mean ± SD)	11,395 ± 13,884	14,690 ± 7569	12,057 ± 7662		
IQR	5220–9049	8984–19,408	6964–15,575	0.025^a^	0.237
CRP (mg/dL, mean ± SD)	1.86 ± 1.47	5.65 ± 6.68	8.09 ± 8.60		
IQR	1.00–2.68	1.00–8.03	1.30–15.08	0.114	0.091
Hospitalization					
YES (n, %)	7 (88 %)	84 (76 %)	14 (64 %)	0.680	0.374
ICU admission					
YES (n, %)	2 (25 %)	9 (8 %)	1 (5 %)	0.162	0.166

*p-value for *S. pneumoniae*, compared to *M. catarrhalis,* **p-value for *H. influenzae*, compared to *M. catarrhalis*

^a^indicates significant (*p* < 0.05)

^b^include bronchitis (*n* = 3)

^c^include pyothorax (*n* = 2), spontaneous bacterial nephritis (*n* = 2), periorbital cellulitis (*n* = 2), arthritis (*n* = 1), mastoiditis (*n* = 1), infective endocarditis (*n* = 1), and infectious pericarditis (*n* = 1)

^d^include periarthritis (*n* = 1), urosepsis (*n* = 1), buccal cellulitis (*n* = 1), osteomyelitis (*n* = 1), and periorbital cellulitis (*n* = 1)

^e^Antimicrobial combination therapy was empirically initiated in a part of patients. (10 in *S.pneumoniae* group and 6 in *H.influenzae*, group)

^f^include ampicillin and amoxicillin

^g^include ampicillin/sulbactam, amoxicillin/clavulanate, piperacillin/tazobactam, and cefoperazone/sulbactam

^h^include cefotiam and cefaclor

^i^include cefotaxime, ceftriaxone, and cefditoren pivoxil

^j^include piperacillin, cefazolin, cefepime, clindamycin, vancomycin, meropenem, panipenem/betamipron, amikacin

Abbreviations: interquartile range, IQR; 7-valent pneumococcal conjugate vaccine, PCV7; 13-valent pneumococcal conjugate vaccine, PCV13; *Haemophilus influenzae* type b, Hib; white blood cell count, WBC; absolute neutrophil count, ANC; C-reactive protein, CRP; intensive care unit, ICU; not available, N/A

### M. catarrhalis

There were eight cases with bacteremia due to *M. catarrhalis*. Mean age was 16 months and six (75 %) were female. Majority of the patients (7/8, 88 %) had underlying diseases. All but one patient were immunocompetent. Characteristically, six patients (75 %) had medical device, including trans-nasal devices (5/8, 63 %). Trans-nasal devices included nasogastric tube (*n* = 3), elemental diet tube (*n* = 2) and nasotracheal tube (*n* = 1). None of the patients was on antibiotics prior to the onset of illness. Six patients (75 %) developed hospital acquired infection and two (25 %) were admitted to the pediatric intensive care unit (PICU). Clinical characteristics of the patients with bacteremia caused by *M. catarrhalis* are shown in detail in Table 2. The focus of infection included pneumonia (*n* = 4) and bronchitis (*n* = 3). Two out of eight patients had concomitant infection with other pathogens; rotavirus gastroenteritis (*n* = 1) and respiratory syncytial virus infection (*n* = 1). All isolated strains of *M. catarrhalis* were beta-lactamase producers. All but one patient were empirically treated with appropriate antibiotics for *M. catarrhalis* (cefotaxime [*n* = 2], ampicillin/sulbactam [*n* = 2], ceftriaxone [*n* = 1], piperacillin/tazobactam [*n* = 1], cefotiam [*n* = 1], and vancomycin [*n* = 1]). The patient empirically treated with vancomycin (for just one day) was switched to cefotaxime soon after the identification of positive blood culture. Three patients were switched to oral antibiotics. The median duration of treatment was 14 days (range: 13–14). All patients recovered without any recurrence or death after the completion of antimicrobial therapy.

**Table 2:** Clinical characteristics of the patients with *Moraxella catarrhalis* bacteremia

	Gender	age (month)	BW (kg)	Underlying diseases	IS	Devices	Sick contact	Symptoms	Pre-existing antibiotics	Focus of infection	Setting	ICU admission	Others
1	female	5	8.3	Biliary atresia	-	YES (ED tube)	NO	Rhinorrhea Cough	None	Bronchitis	Hospital	NO	-
2	female	7	3.1	ELBWI, CLD Multiple malformation	-	NO	YES	Cough	None	Pneumonia	Hospital	YES	Pneumothorax
3	female	8	9.0	Bilateral hydronephrosis	-	NO	YES	Vomiting, Diarrhea	None	Unknown	Community	NO	Rotavirus (+)^a^
4	male	9	7.7	none	-	YES (ETT, NG tube)	NO	Cough	None	Bronchitis	Community	NO	RSV (+)^b^
5	female	15	10.0	Glycogen storage disease type 1b	TAC mPSL	YES (ETT, NG, CVC A line)	NO	-	None	Pneumonia	Hospital	YES	-
6	female	18	6.7	VACTER association, ELBWI, CLD, cAVB, DORV/PS/MS	-	YES (ED tube, PM)	NO	Rhinorrhea Cough	None	Pneumonia	Hospital	NO	
7	male	20	8.8	SDH, vascular ring	-	YES (NG tube)	NO	Vomiting, Wheeze Convulsion	None	Bronchitis	Hospital	NO	-
8	female	44	12.9	Congenital intestinal motor dysfuntion	-	YES (CVC)	NO	Cough	None	Pneumonia	Hospital	NO	-

^a^indicates concomitant infection with rotavirus diagnosed by rapid antigen test

^b^indicates concomitant infection with respiratory syncytial virus by rapid antigen test

*Abbreviations*: *BW* body weight, *ELBWI* extremely low birth weight infant, *CLD* chronic lung disease, *VACTER* Vertebral-Anal-Cardiac-Tracheoesophageal fistula-Renal, *cAVB* complete atrioventricular block, *DORV* double outlet right ventricle, *PS* pulmonary stenosis, *MS* mitral stenosis, *SDH* subdural hemorrhage, *TAC* tacrolimus, *mPSL* methyl prednisolone, *ED* elemental diet, *ETT* endotracheal tube, *NG* nasogastolic, *PM* pacemaker, *CVC* central venous catheter, *A line* arterial line, *ICU* intensive care unit, *RSV* respiratory syncytial virus

### S. pneumoniae

There were 110 cases (105 patients) with bacteremia due to *S. pneumoniae*. Mean age was 35 months and 51 (46 %) were female. Of all patients included, only 26 patients (24.8 %) had received PCV 7 and /or PCV13. Thirty-nine (35 %) patients had underlying disease or condition such as malignancy (*n* = 7), post liver transplantation (*n* = 3), congenital heart diseases (*n* = 3), asplenia (*n* = 2) and cerebrospinal fluid leak (*n* = 1). There were 52 penicillin susceptible *S. pneumoniae* isolates, 22 penicillin intermediate isolates and 36 penicillin resistant isolates. The focus of infection was pneumonia (*n* = 19), otitis media (*n* = 7), sinusitis (*n* = 6) and meningitis (*n* = 4), but the majority had no apparent focus and was classified as occult bacteremia (*n* = 40). Of the 84 hospitalized patients (76 %), nine (8 %) were admitted to PICU. Twenty-one patients (24 %) were treated at the outpatient clinic. Empiric antimicrobial therapy is detailed in Additional file 1: Table S1. Of all patients with *S. pneumoniae*, two patients had recurrences; one was previously immunocompetent with congenital cytomegalovirus infection who developed four episodes of bacteremia, and the other was immunocompromised with medulloblastoma who received surgical, chemo-radiotherapy and peripheral blood stem cell transplantation and had a recurrence of bacteremia two weeks after a treatment with piperacillin (100 mg/kg/day) for 6 days.

### H. influenzae

There were 22 cases with bacteremia due to *H. influenzae*. All but one was caused by *H. influenzae* type b. Mean age was 25 months and 16 (73 %) were male. Of all the patients included with bacteremia due to *H. influenzae*, only one (5 %) had received the Hib vaccine. Nine (41 %) patients had underlying disease or condition such as trisomy 21 (*n* = 2), asplenia (*n* = 1), Cornelia-de-Lange syndrome (*n* = 1), Prader-Willi syndrome (*n* = 1), asthma (*n* = 1), leukemoid reaction with unknown cause (*n* = 1), anterior urethral valve with vesicoureteral reflux (Grade III) and neurogenic bladder (*n* = 1), as well as swallowing dysfunction and rhinopharynx reflux with upper airway stricture (*n* = 1). No patient received immunosuppressants or had medical device. Bacteremia was due to non-typeable *H. influenzae* in one case and the rest were due to Hib. Common sites of infection were the central nervous system (meningitis [*n* = 8]) and lungs (pneumonia [*n* = 5]). Of the 14 (64 %) patients hospitalized, one patient with meningitis was admitted to PICU. Empiric antimicirobial therapy is detailed in Additional file 1: Table S1. The rate of initiating combination antibiotic therapy was highest (27 %) among three groups.

### Comparison of patients’ characteristics

1. *M. catarrhalis* versus *S. pneumoniae*
  The results of univariate analysis comparing the characteristics of patients with bacteremia due to *M. catarrhalis* (*Moraxella* group) and those with bacteremia due to *S. pneumoniae* (Pneumococcus group) are shown in Table 1. *Moraxella* group had lower body weight, higher rate of patients with underlying diseases or conditions and medical devices, particularly trans-nasal devices, compared to the pneumococcus group (*P* = 0.016, *P* = 0.005, *P* = 0.001, and *P* < 0.001, respectively). In addition, a higher rate of disease onset during hospitalization and lower white blood cell count were noted in the *Moraxella* group compared with the pneumococcus group (*P* < 0.001, *P* = 0.011, respectively). A higher rate of patients was on antibiotics in the pneumococcus group (*P* = 0.049). The percentage of patients with a history of contact with sick people and symptoms at the time of onset of illness were not different between the two groups (*P* = 0.472, *P* = 0.281 [for rhinorrhea], *P* = 0.274 [for cough], *P* = 0.445 [for convulsion], *P* = 0.296 [for vomiting], *P* = 0.350 [for diarrhea], respectively). Presence or absence of immunodeficiency and immunosuppressant use was not significantly different between the two groups. Duration of fever after appropriate treatment was approximately one day longer in the *Moraxella* group than in the pneumococcus group (*P* = 0.012).
2. *M. catarrhalis* versus *H. influenzae*
  The results of univariate analyses regarding the relationship between the patients with bacteremia due to *M. catarrhalis* (*Moraxella* group) and those with bacteremia due to *H. influenzae* (*Haemophilus* group) are shown in Table 1. The *Moraxella* group had a higher rate of patients with disease or condition and medical devices, especially trans-nasal devices, compared with the *Haemophilus* group (*P* = 0.039, *P* < 0.001, and *P* < 0.001, respectively). In addition, a lower percentage of the patients were on antibiotics prior to presentation and a higher percentage of those with history of intubation were noted in the *Moraxella* group compared with the *Haemophilus* group (*P* = 0.029, *P* = 0.032, respectively).

## Discussion

Our study demonstrated that patients with bacteremia due to *M. catarrhalis* were more likely to have underlying diseases or conditions, which necessitated the use of medical devices, such as trans-nasal devices, in comparison with those with bacteremia due to *S. pneumoniae* or *H. influenzae*. To the best of our knowledge, this is the first report that describes the differences in background and clinical characteristics of *M. catarralis* bacteremia compared with pneumococcal and *H. influenzae* bacteremia.

*M. catarrhalis* is regarded as one of the most common bacterial causes of respiratory tract infections, following *S. pneumoniae* and *H. influenzae*. According to reports from 1992 to 2006, 15 − 20 % of acute otitis media episodes were caused by *M. catarrhalis* on the basis of cultures of middle ear fluid obtained by tympanocentesis [7, 16–18]. Colonization rate of *M. catarrhalis* in Japanese children attending day-care centers was 34.6 %, which followed *S. pneumoniae* (60.3 %) and *H. influenzae* (53.2 %) in the pre-vaccine era [19]. Several reports from countries where PCVs are widely used demonstrated that colonization by vaccine serotypes of pneumococcus were decreasing, while colonization by the pneumococcus with non-PCV serotypes *H. influenzae* and *M. catarrhalis* were increasing, resulting in a change in the pathogens that cause otitis media [7, 20, 21] and sinusitis [7, 22, 23]. However, with the recent inclusion of Hib vaccine and PCV [13] in the national immunization program in Japan, the colonization rate of *M. catarrhalis* may increase in the near future.

*M. catarrhalis* is an important pathogen in respiratory tract infections in children, but bacteremia is rare [24]. Several recent reports revealed that previously healthy immunocompetent patients developed *Moraxella* bacteremia at a constant rate [24–27]. In the current study, however, only one previously healthy child developed bacteremia due to *M. catarrhalis* and the remaining seven patients had underlying disease. A previous report described 11 cases of bacteremia due to *M. catarrhalis* and noted that it should be considered in febrile children with underlying immunodeficiency and upper respiratory tract infection [8]. Ioannidis et al. noted that nearly 30 % of patients with *M. catarrhalis* bacteremia had underlying respiratory factors including tracheostomy or dependence on mechanical ventilation, and acute respiratory infections such as viral pneumonia [28]. The authors also described an association with chronic neurological debilities as one of the predisposing factors [28]. Similarly, we found that a quarter of the patients had underlying respiratory diseases, and three out of eight patients had chronic neurological problems. More specifically, our study highlights the association of medical devices, particularly trans-nasal devices, as independent factors associated with bacteremia due to *M. catarrhalis* compared with *S. pneumoniae* or *H. influenzae*. Most isolates of *M. catarrhalis* produce beta-lactamases in contrast to *S. pneumoniae* which acquire modest ampicillin resistance through modification of the penicillin binding proteins. Our findings suggests that modification of empiric coverage to cover penicillinase producing *M. catarrhalis* is a consideration for patients with underlying disorders with trans-nasal devices who develop respiratory tract infections.

The pathogenic association of medical devices and invasive disease caused by *M. catarrhalis* is unclear. The nasopharyngeal colonization of *M. catarrhalis* is quite common among children, especially through infancy, and *M. catarrhalis* forms biofilms and has been identified in the middle ears of children with otitis media [7]. Adherence of *M. catarrhali*s to the respiratory epithelium and mucin molecules of the nasopharynx and the middle ear is known to be a key step in the initiation of infection [29, 30]. *M. catarrhali*s has also been found intracellularly in subepithelial cell and lymphoid tissue [31], with a possibility that the breach in the integrity of the mucosal barrier by medical devices may also contribute to the development of invasive infections. A previous report identified the presence of endotracheal tubes and frequent endotracheal tube suctioning as risk factors for developing nosocomial infection due to *M. catarrhalis*, which support our findings [32]. Moreover, another report describes the association of respiratory tract fragility with bacteremic pneumonia caused by *M. catarrhalis* may reflect increased rates of oropharyngeal colonization, enhancement of bacterial adherence to abnormal epithelium, and increased susceptibility of pulmonary parenchyma to infection [28, 33]. According to a study using an animal model, type IV pili of *M. catarrhalis* plays a crucial role in colonization of the nasopharyngeal mucosa and biofilm formation [34]. In addition, the biofilms produced by *M. catarrhalis* are similar to the complicated structures produced by several bacteria, such as *Pseudomonas aeruginosa*. Biofilm formation is important in considering infections associated with foreign body, such as endotracheal tube [34, 35]. However, it has yet to be revealed whether biofilms contribute to pathogenesis of infections caused by *M. cararrhalis*. Further basic and clinical studies are needed to clarify our hypothesis.

A previous report that described the clinical and laboratory aspects of *M. catarrhalis* bacteremia in 17 children showed that most patients with *M. catarrhalis* bacteremia had modest elevation in body temperature (mean ± SD: 37.3 ± 1.2 °C) and leukocytosis (mean ± SD: 12,070 ± 7569 /μL) [24]. In the current study, although all patients had a maximum temperature >38.0 °C, the fever tended to be mild and leukocyte count was 14,774 ± 13,750/μL (mean ± SD), denoting similar tendency with the previous report. Such data suggests that *M. catarrhalis* is associated with relatively mild inflammation. However, the patients’ immune statuses including neutrophil function, complement and humoral immunity, which may influence the degree of inflammation, were not fully investigated in the current study.

We acknowledge that there are several limitations to this study. First, this study is a retrospective case–control-control study based on a single center experience in Japan. In addition, there were few patients with *M. catarrhalis* bacteremia. Thus, we performed only univariate analyses, and therefore our results should be interpreted carefully. Second, this study was conducted on patients with bacteremia due to *H. influenzae* and *S. pneumoniae* during a transition period of the national immunization program in Japan before and after introduction of Hib vaccine and PCV, which may influence patient background. Finally, the study evaluated the clinical characteristics of the *Moraxella* bacteremia patients against those with pneumococcal or *Haemophilus* bacteremia. Therefore, our study is likely to underestimate the risk factors that are common to the development of bacteremia caused by all three organisms. Hereafter, collection and evaluation of national surveillance data regarding *Moraxella* bacteremia patients are required to clarify epidemiological changes in the post-vaccine era and the risk factors of patients with *Moraxella* bacteremia.

## Conclusions

Patients with bacteremia due to *M. catarrhalis* were likely to have underlying diseases or conditions requiring medical devices, particularly trans-nasal devices.

## Additional file

Additional file 1: Table S1. Empiric antimicrobial therapy among three groups. (DOCX 35 kb)

## Abbreviations

Hib: *H. influenzae* type b
IHD: Invasive haemophilus disease
IPD: Invasive pneumococcal diseases
NCCHD: National Center for Child Health and Development
PCV: Pneumococcal conjugate vaccine
PICU: Pediatric intensive care unit.
SD: Standard deviation
