# Supplementary material for: Clinical characteristics of the patients with bacteremia due to Moraxella catarrhalis in children: a case–control study
Source: BMC Infect Dis. 2016 Feb 9;16:73. doi: 10.1186/s12879-016-1408-3 (PMC4748453; doi:10.1186/s12879-016-1408-3)
Supplement: Additional file 1: Table S1. — Empiric antimicrobial therapy among three groups. (DOCX 35 kb) [file 12879_2016_1408_MOESM1_ESM.docx]

**Supplementary table 1**. Empiric antimicrobial therapy among three groups

| Antimicrobial therapy | *M. catarrhalis*  (n = 8) | *S. pneumoniae*  (n = 110) | *H. influenzae*  (n = 22) |
| --- | --- | --- | --- |
| MONOTHERAPY | | | |
| Ampicillin | 0 | 16 | 0 |
| Amoxicillin | 0 | 2 | 0 |
| Ampicillin/sulbactam | 2 | 5 | 1 |
| Amoxicillin/clavulanate | 0 | 1 | 0 |
| Piperacillin | 0 | 3 | 0 |
| Piperacillin/tazobactam | 1 | 1 | 0 |
| Cefoperazone/sulbactam | 0 | 1 | 0 |
| Cefazolin | 0 | 1 | 2 |
| Cefotiam | 1 | 1 | 0 |
| Cefaclor | 0 | 1 | 0 |
| Cefotaxime | 2 | 43 | 10 |
| Ceftriaxone | 1 | 18 | 1 |
| Cefditoren pivoxil | 0 | 2 | 0 |
| Cefepime | 0 | 1 | 0 |
| Meropenem | 0 | 0 | 0 |
| Panipenem/betamiprone | 0 | 0 | 0 |
| Vancomycin | 1 | 0 | 0 |
| Clindamycin | 0 | 1 | 0 |
| COMBINATION THERAPY | | | |
| The rate of combination therapy | 0 (0%) | 10 (9%) | 6 (27%) |
| Cefotaxime + vancomycin | 0 | 7 | 2 |
| Ceftriaxone + vancomycin | 0 | 1 | 0 |
| Cefepime + vancomycin | 0 | 1 | 0 |
| Cefotaxime + meropenem | 0 | 0 | 1 |
| Cefotaxime  + panipenem/betamiprone | 0 | 0 | 2 |
| Ceftriaxone + clindamycin | 0 | 0 | 1 |
| Ampicillin + amikacin | 0 | 1 | 0 |
| NO TREATMENT | 0 | 3 | 2 |
